# Generation of Chimera-Competent Avian iPSCs Using Defined Transcription Factors

**DOI:** 10.3390/cells15121092

**Published:** 2026-06-16

**Authors:** Xinyi Tong, Xi Chen, Arlene Anicete, Yanpui Chan, Xuan Zhou, Xizi Wang, Daniel B. McKim, Qi-Long Ying

**Affiliations:** Eli and Edythe Broad Center for Regenerative Medicine and Stem Cell Research at USC, Department of Stem Cell Biology and Regenerative Medicine, Keck School of Medicine, University of Southern California, Los Angeles, CA 90033, USA; xinyit@usc.edu (X.T.); xichen@caltech.edu (X.C.); anicete@usc.edu (A.A.); yanpuich@usc.edu (Y.C.); xzhou595@usc.edu (X.Z.); wangxizi1018@gmail.com (X.W.); dmckim@usc.edu (D.B.M.)

**Keywords:** avian iPSCs, reprogramming, stem cell self-renewal

## Abstract

iPSC technology is well established in mammals but remains underdeveloped in non-mammalian species. A major barrier to generating avian iPSCs has been the lack of species-specific reprogramming factors and culture conditions capable of supporting self-renewal in avian pluripotent stem cells. Here, we report the generation of chicken iPSCs (ciPSCs) using a cocktail of seven chicken transcription factors (T7: Oct4, Sox2, Sox3, Klf4, c-Myc, Nanog, and Lin28B) combined with an optimized avian culture system. Transcriptomic and functional analyses identified Sox3, rather than Sox2, as the predominant SoxB1 factor in avian reprogramming. The resulting ciPSCs exhibited stable self-renewal for over 40 passages, expressed core pluripotency markers, differentiated into all three germ layers, and were transcriptionally similar to chicken ESCs. In chimera assays, ciPSCs contributed to somatic, extra-embryonic, and germline lineages, giving rise to gonadal PGC-like cells that did not acquire full germline competence. We further demonstrate that the T7 system generates iPSCs from quail, duck, peacock, zebra finch, and pigeon, and that duck iPSCs can form interspecies chimeras with donor cells detected in the host gonads. These findings establish a generalizable platform for avian iPSC generation with applications in developmental biology and germline preservation of endangered species.

## 1. Introduction

Since the landmark discovery by Yamanaka and colleagues in 2006, induced pluripotent stem cells (iPSCs) have transformed stem cell biology by demonstrating that differentiated somatic cells can be reprogrammed back to a pluripotent state. Like embryonic stem cells (ESCs), iPSCs possess the capacity for long-term self-renewal and differentiation into derivatives of all three germ layers, making them powerful tools for a wide range of applications [1,2,3]. Over the past two decades, iPSCs have been widely used as in vitro platforms for studying early embryonic development, lineage specification, disease modeling, regenerative medicine, and evolutionary and developmental biology. In addition to these biomedical and basic research applications, iPSCs also hold great promise for species conservation. Unlike ESCs, which depend on embryo-derived materials, iPSCs can be generated directly from differentiated somatic cells, providing a more accessible and ethically acceptable source of pluripotent cells, particularly for endangered species for which embryos are difficult or impossible to obtain [4]. In recent years, iPSC technology has been successfully applied to several endangered mammals, including the giant panda and the northern white rhinoceros, highlighting its potential for germline preservation, genetic rescue, and biodiversity conservation [5,6,7,8]. Despite these broad opportunities, iPSC research in non-mammalian species remains limited, particularly in birds [9].

Several groups have generated chicken iPSCs (ciPSCs) by reprogramming chicken embryonic fibroblasts (CEFs). Early studies showed that induced expression of four mammalian transcription factors, OCT4, KLF4, SOX2, and c-MYC, known as OSKM, could induce iPSC-like colonies in chicken cells [10]. Subsequent work using chicken homologs of OKSM demonstrated that the additional inclusion of NANOG and LIN28 improved reprogramming efficiency [11]. Similarly, fusion of the MyoD-derived transactivation domain to OCT3/4 (M3O) was also reported to enhance reprogramming efficiency [12,13]. However, none of these studies succeeded in establishing long-term cultured ciPSCs capable of sustained expansion while retaining germline competence [11,14,15,16]. Moreover, none of the previously reported ciPSC lines generated highly integrated chimeras, highlighting the incomplete functional pluripotency of these previously described cells. A major barrier has been the lack of culture conditions that can support stable self-renewal and preserve pluripotency in avian cells.

A parallel challenge has existed for chicken ESCs (cESCs). Although chicken blastodermal cells contain germline-competent pluripotent cells, previously established cESC-like lines tend to lose their capacity for chimera formation and germline transmission after prolonged culture [17,18,19]. Our laboratory recently addressed this problem by developing defined culture conditions containing ovotransferrin (OT), chicken leukemia inhibitory factor (chLIF), and three small molecule inhibitors (IWR-1, Gö6983, SB431542), collectively referred to as OT/3i/chLIF. This system supported the derivation and long-term maintenance of germline-competent cESCs, and the underlying self-renewal pathways proved to be conserved across multiple avian species [20]. In addition, we developed a minimal, serum-free basal medium supplement to replace conventional N2B27, which was originally formulated for neuronal culture and contains components that can promote unwanted lineage commitment cells [21]. The minimal supplement contained only four components: insulin, transferrin, BSA, and selenite, and was termed “E4”. We reasoned that adapting these optimized culture conditions for avian cells, combined with the correct species-matched reprogramming factors, would be critical for generating functional avian iPSCs. Here, we report the generation of stable, long-term expandable ciPSCs by combining an optimized culture system with a defined cocktail of seven chicken transcription factors. We demonstrate that these ciPSCs exhibit functional pluripotency and contribute extensively to somatic chimeric embryos. We further show that this reprogramming strategy is broadly applicable across diverse avian species, providing a versatile platform for studying avian pluripotency and for germline preservation of endangered birds.

## 2. Materials and Methods

### 2.1. Avian Animals and Fertile Eggs

All animal procedures were carried out in accordance with protocols approved by the University of Southern California Institutional Animal Care and Use Committee.

Fertile avian eggs used for embryonic fibroblasts derivation were purchased from the following sources: Roslin Green GFP Eggs were purchased from Clemson University, SC, USA. Specific-pathogen-free fertile chicken eggs (Charles River Laboratories, MA, USA), Rhode Island Red chicken (AA lab eggs, Westminster, CA, USA), Japanese quail and Duck eggs (AA Lab Eggs, Westminster, CA, USA), zebra finch eggs (Dr. Carlos Lois’ lab at the California Institute of Technology, CA, USA), peacock (Ebay Inc., CA, USA).

### 2.2. Feeder Cells Preparation

CF-1 mouse embryonic fibroblasts (MEFs) were used as feeder cells to support the growth of pluripotent stem cells. CF-1 strain mice were purchased from Charles River. CF-1 MEFs were isolated from an E13.5 embryo and expanded to ~80–90% confluence in DMEM supplemented with 10% FBS, 1% non-essential amino acids, and 1% penicillin–streptomycin. Cells were harvested using 0.05% trypsin–EDTA, washed with PBS, and resuspended at a density of 1–2 × 10^7^ cells/mL. To inactivate the feeders, MEFs were exposed to X-irradiation at 5000 rad using an irradiator(XRad320, PRECISION X-Ray Inc., CT, USA). Immediately after irradiation, cells were plated onto 0.1% gelatin-coated culture dishes at a density of 2–3 × 10^4^ cells/cm^2^ and allowed to attach overnight before use. Irradiated feeders remained viable but non-proliferative for 5–7 days and were prepared fresh weekly to ensure optimal support for pluripotent stem cell cultures.

### 2.3. CEF Derivation

Chicken embryonic fibroblasts (CEFs) were derived from E10.5 chicken embryos. Briefly, chicken embryos were rinsed with PBS, and heads, visceral tissues, and limbs were removed. The remaining bodies were minced using sterile scissors, rinsed with PBS, and then incubated in 0.25% Trypsin-EDTA (Gibco, Thermo Fisher Scientific, MA, USA) at 38 °C for 5–7 min. Tissues were gently vortexed once during the dissociation process. Following trypsinization, the digested tissues were neutralized with DMEM supplemented with 10% FBS (DF) and filtered through a 70 µm Nylon cell strainer (Corning Inc., NY, USA) before centrifugation. The resulting cell pellet was resuspended and cultured in DF medium. One to two days prior to transduction, CEFs were dissociated using 0.25% Trypsin-EDTA (Gibco) for 3 min at 38 °C and passaged into a new culture plate.

### 2.4. Plasmid Constructs and Retrovirus Production

Murine leukemia virus (MLV)–based retroviral vectors derived from pMX-GFP were used as backbones to express the different transcription factors. All transcription factor cDNAs were either commercially synthesized or PCR-amplified from cDNA reverse-transcribed from RNA isolated from chicken embryonic stem cells. Retroviral particles were produced by transfection of Lenti-X 293T cells cultured in 100 mm dishes, using pMX-based expression plasmids together with the packaging plasmids PLK and HMDG, and polyethylenimine (PEI 25,000; Polysciences Inc., PA, USA) as the transfection reagent. The culture medium was replaced with fresh DF medium the following day. Viral supernatants were collected 48 h after transfection and concentrated using the Lenti-X^TM^ Concentrator (Takara Bio USA, Inc., CA, USA) according to the manufacturer’s instructions.

### 2.5. AC Medium Preparation for Routine Avian iPSC Culture

The previously established OT/3i/chLIF culture system, developed for chicken ESC derivation and maintenance [20], required two key modifications for iPSC reprogramming of chicken embryonic fibroblasts (CEFs). SB431542, one of the three small molecule inhibitors (3i) in the original formulation, was toxic to CEFs and reduced reprogramming efficiency (Supplementary Figure S1A). It was replaced with CP-673451, a selective PDGFR inhibitor that had been identified in parallel work as a stabilizer of chicken ESC self-renewal during long-term culture [20]. CP-673451 supported robust colony formation without the toxicity observed with SB431542.

The N2B27 basal medium was also replaced. N2B27 was originally formulated for neuronal culture and contains multiple additives beyond what is required for pluripotent cell maintenance [22]. A systematic screen of N2B27 components had identified only four as essential for ESC self-renewal: insulin, transferrin, BSA, and sodium selenite [21]. This minimal formulation, termed E4, excludes pro-neural additives that may promote unwanted differentiation. For avian cells, E4 was further modified by substituting BSA with lipid-rich Albumax. Human transferrin was also replaced with ovo-transferrin for species compatibility. Human insulin was retained in the AC medium because insulin signaling is highly conserved across vertebrates, and recombinant chicken insulin is not commercially available. By contrast, components that showed clear species-specific effects were replaced, namely ovo-transferrin and chicken LIF. Colonies generated under the optimized E4 conditions with CP-673451 showed improved morphology compared with those maintained in the original N2B27-based formulation, and SB431542 became dispensable (Supplementary Figure S1B). The resulting formulation, termed AC medium (E4 basal medium with IWR-1, Go6983, CP-673451, and chLIF), was used for all subsequent reprogramming experiments. The basal medium for maintaining avian iPSCs (AC medium) consisted of E4 medium, formulated by combining DMEM/F12 (Gibco, 12400024) and Neurobasal medium (Gibco, 21103049) at a 1:1 ratio. The following supplements were added: human insulin (4 µg/mL; Sigma, MO, USA. 91077C-250MG), sodium selenite (12.5 ng/mL; Sigma, S9133-1MG), ovo-transferrin (10 µg/mL; Sigma, C7786), and 0.25% lipid-rich Albumax (Gibco, 11020021). The medium was further supplemented with 1× penicillin–streptomycin. To generate the complete AC medium used for iPSC maintenance, E4 medium was supplemented with 2.5 µM IWR-1 (Selleckchem Chemicals, TX, USA), 2 µM Go6983 (Selleckchem Chemicals, TX, USA), 1 µM CP-673451 (Selleckchem Chemicals, TX, USA), and 10 ng/mL recombinant chicken LIF (Kingfisher Biotech Inc., MN, USA). All cultures were maintained at 38 °C with 5% CO_2_ unless otherwise specified.

### 2.6. Avian iPSC Generation and Maintenance

On the day of transduction (Day 0), when CEFs reached 70–90% confluency, cells were transduced with retroviruses encoding Oct4, Sox2, Sox3, Klf4, c-Myc, Nanog, and Lin28B in the presence of 8 µg/mL polybrene (Sigma-Aldrich, MO, USA). On Day 1, transduced CEFs were passaged and cultured in DF medium for expansion. On Day 2, the culture medium was switched to AC medium (E4/3i/cLIF), which was subsequently replaced every other day. iPSC-like colonies typically appeared around Day 6 post-transduction. Once distinct colonies became visible, they were manually picked and reseeded onto irradiated feeder cells for continued expansion. Avian iPSCs were maintained at 38 °C in 5% CO_2_ with daily medium changes, and all other avian iPSC lines were derived using the same procedure as for chicken iPSCs.

For passaging, cultures were washed once with 1× PBS and incubated with 0.025% trypsin for ~3 min at room temperature, followed by neutralization with twice the volume of DF medium (DMEM supplemented with 10% FBS). Cell clumps were gently dissociated using 1 mL pipette tips to minimize mechanical damage. For cryopreservation, iPSCs were resuspended in Cellbanker 2 (Amsbio, MA, USA) and stored at −150 °C.

### 2.7. Quantitative Real-Time PCR (qRT-PCR)

RNA was extracted and purified using the RNeasy Mini Kit (QIAGEN, MA, USA). 1 µg of total RNA was reverse-transcribed into cDNA using iScriptTM Reverse Transcription Supermix (BIO-RAD, CA, USA). qRT-PCR was performed using the iTaqTM Universal SYBR^®^ Green Supermix (BIO-RAD) on an Applied Biosystems QuantStudioTM 7 Flex Real-Time PCR machine (Applied Biosystems, CA, USA). Primer sequences are listed in Table S1.

### 2.8. RNA-Seq Sample Preparation and Analysis

Chicken EGK.X stage embryos were harvested using the filter paper-based method. Total RNA from chicken iPSCs, ESCs, and EGK.X embryos was extracted using the RNeasy Mini Kit (Qiagen, MA, USA) according to the manufacturer’s instructions. RNA samples were sent to Novogene for library preparation and high-throughput sequencing. Only RNA samples that passed Novogene’s quality-control criteria for eukaryotic mRNA-seq, including RIN ≥ 4.0 with a flat baseline, were used for library preparation. For each cell type, two biological replicates were collected, and a minimum of 20 million paired-end reads was generated per replicate. For each sample type, two biological replicates were collected and analyzed. Messenger RNA was purified from total RNA using poly-T oligo-attached magnetic beads. Following fragmentation, first-strand cDNA was synthesized using random hexamer primers, followed by second-strand cDNA synthesis. Sequencing libraries were then generated through end repair, A-tailing, adapter ligation, size selection, amplification, and purification. Library quality was assessed using Qubit and real-time PCR for quantification, and an Agilent Bioanalyzer was used to determine library size distribution.

The quantified libraries were pooled and sequenced by Novogene on an Illumina NovaSeq X Plus platform using a 150 bp paired-end read configuration. A minimum of 20 million paired-end reads, corresponding to approximately 6 Gb of raw sequencing data, was generated for each replicate. Downstream RNA-seq data analysis was performed using the Partek Flow version 12.10.0 platform.

Raw sequencing data were processed and analyzed using Partek Flow. Reads were aligned to the chicken reference genome (GRCg6a, Ensembl) using STAR, and gene-level read counts were quantified with RSEM. Differential expression analysis was performed with DESeq2, defining significantly regulated genes as those with |log2 fold change| > 1, adjusted *p*-value < 0.05, and baseMean > 200. For downstream visualization, count data were transformed using the regularized logarithm (rlog) function in DESeq2, and principal component analysis (PCA) and unsupervised hierarchical clustering were carried out. Plots, including PCA and heatmaps, were generated using ggplot2 and ComplexHeatmap in R.

### 2.9. In Vitro Differentiation of ciPSCs

ciPSCs were differentiated through embryoid body (EB) formation. Briefly, ciPSCs were dissociated with 0.025% trypsin and resuspended in N2B27 basal medium supplemented with lineage-specific factors. Cells were seeded into AggreWell™ 400 24-well plates (STEMCELL Technologies, Canada) at approximately 150,000 cells per well. After suspension culture, EBs were plated onto coated wells for adherent differentiation. Lineage marker expression was assessed by immunostaining and qRT-PCR.

For neural differentiation, EBs were formed overnight in AggreWell™ 400 plates and then transferred to non-adherent wells for an additional 3 days in N2B27 medium supplemented with 2 µM IWR-1, 2.5 µM Gö6983, and 2 µM SB431542. The medium was then changed to N2B27 containing 5% chicken serum for 7 days. EBs were subsequently plated onto Matrigel-coated 4-well plates and cultured for 2 additional days before immunostaining.

For muscle differentiation, ciPSCs were resuspended in N2B27 medium supplemented with 15 ng/mL bFGF, 3 µM CHIR, and 100 nM LDN193189 for EB formation. After 5 days of suspension culture, EBs were plated onto Matrigel-coated wells and cultured in N2B27 medium for an additional 4 days before immunostaining.

For endoderm differentiation, ciPSCs were resuspended in N2B27 medium supplemented with 15 ng/mL bFGF for EB formation. After 5 days of suspension culture, EBs were plated onto 0.1% gelatin-coated wells and cultured for an additional 4 days in N2B27 medium containing 5% chicken serum before immunostaining.

### 2.10. Ex Ovo Chimera Formation Assay

Albumen agar plates containing 0.3% agarose, 0.3% glucose, 20 U/mL penicillin, and 20 µg/mL streptomycin were freshly prepared for embryo culture. Freshly laid eggs were opened into Petri dishes, and the thick albumen was removed to expose the blastoderm. Embryos were collected using a filter paper carrier as described in the EC culture system [23]. The filter paper was cut around the embryo, and the embryo was transferred into PBS to remove residual yolk before being placed ventral side up on albumen agar plates.

For chimera formation, GFP-labeled ciPSCs were dissociated with 0.025% trypsin for 3 min at room temperature. After neutralization, cells were treated again with 0.025% trypsin for 6 min at 37 °C to obtain a single-cell suspension. Approximately 20,000 GFP-positive ciPSCs were evenly distributed onto the recipient embryo using a glass microcapillary. Embryos were cultured at 38 °C for 2–4 days, and donor-cell contribution was monitored by live fluorescence imaging.

### 2.11. In Ovo Chimera Formation Assay

Chimeric embryos were cultured in ovo using a two-step surrogate eggshell system. Where indicated, recipient fertile eggs were irradiated at 500 cGy before injection. For the first culture step, the contents of each recipient egg were transferred into a surrogate eggshell weighing 4–5 g more than the original egg. Donor ESCs were dissociated into single cells as described for the ex ovo chimera assay, and 8000 cells in 0.75 µL were injected into the subgerminal cavity using a glass microcapillary. After injection, the egg was supplemented with albumen, sealed with cling film, and secured with PVC plastic rings and rubber bands. The reconstructed eggs were placed window-side up on a tilted shaker at approximately 1 rpm overnight, then incubated upside down at 37.5 °C with 90° rocking at 1 h intervals. After 3–4 days, chimerism was assessed by live GFP imaging. For continued development, embryos were transferred into jumbo surrogate shells weighing 20–25 g more than the original recipient eggs. The shells were resealed with cling film and incubated at 37.5 °C until hatching.

### 2.12. Chicken PGCs Derivation

Fertilized chicken eggs were incubated at 38 °C in a humidified incubator until either the blood-borne PGC stage (Hamburger–Hamilton stage 14–16) or the early gonadal stage (embryonic day 5.5–7). For blood-derived PGCs, the dorsal aorta was punctured with a fine glass capillary to collect circulating cells into chicken PGCs basal medium. For gonadal PGCs, paired gonads were dissected from the embryonic mesonephros, minced with fine scissors and resuspended in chicken PGCs basal medium. After spinning down, PGCs were plated onto a non-treated plate with FAOT medium developed by the McGrew group [24].

### 2.13. Chicken EGs Converted from PGCs

Chicken primordial germ cells (PGCs) were converted into embryonic germ (EG) cells using culture conditions adapted from our previously established OT/3i/chLIF chicken ESC system [20]. Cultured PGCs were directly plated in chicken ESCs culture conditions with a feeder layer. Cells were maintained at 38 °C in 5% CO_2_, with daily medium changes. PGCs gradually expanded and formed compact colonies over 5–7 days; emerging EG-like colonies were manually picked and transferred to fresh feeders for continued passaging.

### 2.14. Immunostaining

Gonadal tissues were fixed in 4% paraformaldehyde at 4 °C overnight and transferred to 30% sucrose until sinking to the bottom of the vial. Subsequently, tissues were embedded in OCT and cryosectioned for immunofluorescence. For staining of attached cells, cells were fixed in 4% paraformaldehyde for 20 min at room temperature. After fixation, all samples were blocked with PBS containing 5% normal donkey serum and 0.2% Triton X-100 for 1 h. Samples were then incubated with primary antibodies overnight at 4 °C. After three washes in PBS with 0.1% Tween-20, secondary antibodies were applied for 2 h at room temperature. Samples were washed three times in PBS with 0.1% Triton X-100 before mounting for imaging. Nuclei were stained with Hoechst 33342 (Invitrogen, CA, USA 1:50,000). Primary antibodies used include: Myosin (MF-20, DSHB, IA, USA 1:50), TuJ-1 (MAB1195, R&D Systems, MN, USA, 1:100), DAZL (ab215718, Abcam, Cambridge, UK, 1:750), GFP (A11120, Invitrogen, CA, USA, 1:500), Tdtomato (600401379, Rockland, PA, USA, 1:300).

### 2.15. Genomic DNA Extraction and PCR

Genomic DNA was extracted from chicken feathers using the Puregene Cell Kit (Qiagen) according to the manufacturer’s instructions. Briefly, tissues were dissected and subjected to tissue lysis, followed by protein precipitation and DNA precipitation. The DNA pellet was washed, air-dried, and resuspended in nuclease-free water. Approximately 10 ng of genomic DNA was used as a template for each PCR reaction. EGFP integration was assessed using primers specific for the EGFP coding sequence (forward: 5′-GCGCACCATCTTCTTCAAGG-3′; reverse: 5′-GGGGTGTTCTGCTGGTAGTG-3′), yielding a 267 bp amplicon. PCR was performed using 2× Q5 High-Fidelity PCR Master Mix (New England Biolabs, MA, USA) in a total reaction volume of 20–25 µL, under standard cycling conditions recommended by the manufacturer. PCR products were analyzed by agarose gel electrophoresis and visualized under UV illumination to confirm the presence of the expected EGFP band.

### 2.16. Karyotyping

For chicken iPSCs, metaphase spreads were prepared after colcemid and ethidium bromide treatment. G-banding and karyotype analysis were performed at the Molecular Cytogenetics Laboratory, Texas A&M University.

## 3. Results

### 3.1. OSKM Alone Is Not Sufficient for ciPSC Reprogramming

**Figure 1:**
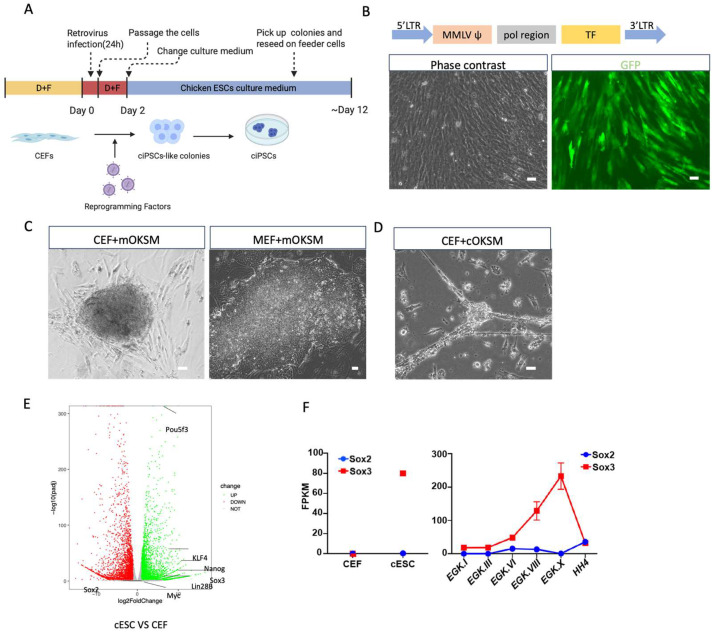
OSKM is insufficient to reprogram chicken embryonic fibroblasts (CEFs) into iPSCs. (**A**) Schematic overview of the experimental design for iPSC reprogramming. CEFs were maintained in DMEM supplemented with 10% FBS (D + F) and transduced with retroviruses encoding different transcription factors. After passaging, the cells were expanded in FBS-containing medium for 2 days, followed by switching to ESC culture medium. Once colonies appeared, they were manually picked and reseeded onto feeder cells; (**B**) Representative image showing chicken embryonic fibroblasts (CEFs) transduced with a GFP-expressing retroviral vector. The pMX-based retroviral vector, derived from the Moloney murine leukemia virus (MMLV), contains a 5′ MMLV long terminal repeat (LTR) promoter driving EGFP expression. Scale bar, 50 µm; (**C**) Representative images of CEFs (**left**) and mouse embryonic fibroblasts (MEFs, **right**) transduced with retroviral vectors carrying the mouse Oct4, Sox2, Klf4, and Myc transgenes (mOSKM). Scale bar, 50 µm; (**D**) Representative image showing CEFs transduced with retroviral vectors carrying chicken Oct4, Sox2, Klf4, and Myc (cOSKM). Scale bar, 50 µm; (**E**) Comparative RNA-seq analysis between chicken ESCs and CEFs identifying differentially expressed genes; (**F**) Expression levels (FPKM) of Sox2 (blue) and Sox3 (red) in chicken ESCs, CEFs, and early-stage embryos (EGK.I to HH4). Data represent mean ± s.d. from two biological replicates. CEF RNA-seq data were obtained from the accession number GSM2735363.

iPSCs have been successfully generated from various mammalian species through the forced expression of OSKM [2,3,25,26]. To determine whether avian iPSCs could be generated under similar conditions, we performed reprogramming experiments using a modified version of our OT/3i/chLIF culture system [20] with an avian-adapted E4 basal medium [21] (see Section 2 and Supplementary Figure S1). The experimental approach to generate ciPSCs is illustrated in Figure 1A, where retroviral transduction is used to transduce chicken embryonic fibroblasts (CEFs). Using retroviral transduction, CEFs from Rhode Island Red embryos were successfully transduced to overexpress GFP (Figure 1B). While the introduction of mouse OSKM successfully reprogrammed mouse embryonic fibroblasts (MEFs) into iPSCs (Figure 1C Left Panel), no expandable colonies were obtained from CEFs following OSKM transduction (Figure 1C Right Panel). Substituting mouse OSKM with the corresponding chicken orthologs also failed to produce chicken iPSCs. Instead, only neuronal-like cells appeared after passaging (Figure 1D). These findings indicate that OSKM alone is insufficient to reprogram CEFs into iPSCs.

### 3.2. Identification of Additional Reprogramming Factors for ciPSCs

To identify factors potentially involved in avian pluripotency, we compared RNA-seq profiles of cESCs and CEFs and identified a set of transcription factors highly expressed in cESCs (Figure 1E). This included three canonical OSKM factors (Oct4/Pou5F3, KLF4, and MYC) but notably not Sox2. Instead, the related SoxB1 family member Sox3 was highly expressed, consistent with previous work suggesting that Sox3, rather than Sox2, is the predominant SoxB1 factor associated with pluripotency in avian species [20,27]. In addition, Nanog and Lin28B, both of which have been previously implicated in somatic cell reprogramming [11,28,29,30], were also enriched. We further analyzed chicken primary cells at early developmental stages (EGK.I to HH4) and found that Sox3 was highly expressed in both chicken ESCs and EGK.X embryos, whereas Sox2 was not significantly enriched (Figure 1F). Together, these expression profiles suggested that Oct4, Sox3, Klf4, Myc, Nanog, and Lin28B represent a rational set of candidates reprogramming factors for generating ciPSCs.

### 3.3. Generation and Characterization of T7-Derived ciPSCs

**Figure 2:**
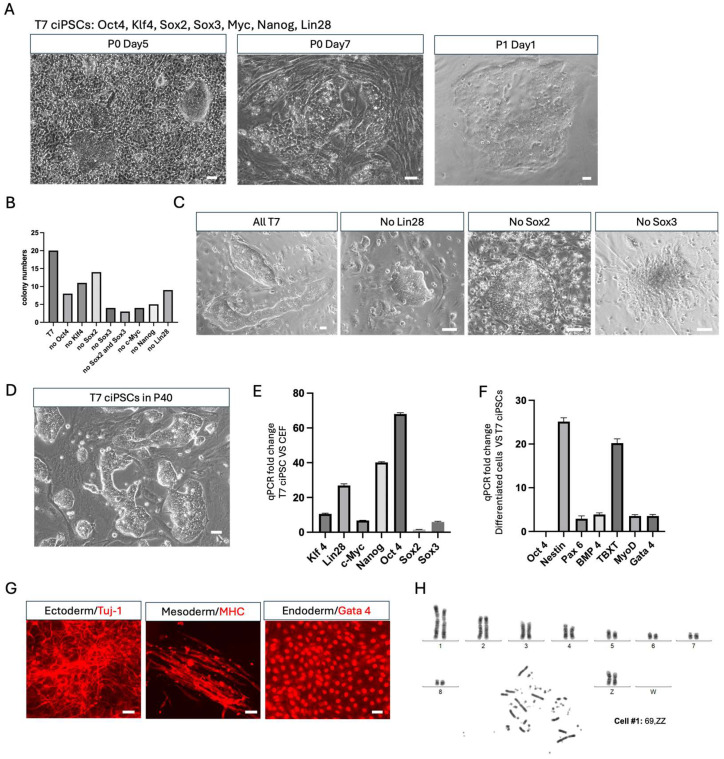
Generation of chicken iPSCs using the T7 transcription factor cocktail. (**A**) Morphological progression of chicken iPSC colony formation by all seven transcription factors (T7: Oct4, Klf4, Sox2, Sox3, Myc, Nanog, Lin28) at different stages: Passage 0 (P0) day 5 (initial colony appearance), Passage 0 (P0) day 7 (colonies ready for picking), and Passage 1 (P1) day 1 (reseeded colonies). Scale bars, 50 µm; (**B**) Quantification of colony numbers generated by different transcription factor combinations. Colonies were counted from two wells of a 6-well plate (1.2–1.5 × 10^6^ CEFs in total) for each condition. Removal of individual factors from the T7 cocktail (Oct4, Klf4, Sox2, Sox3, c-Myc, Nanog, and Lin28B) markedly reduced reprogramming efficiency; (**C**) Representative phase-contrast images of colonies generated under selected conditions: all T7 factors (All), no Lin28B, no Sox2, and no Sox3. Colonies lacking Sox3 displayed neuronal-like morphology, indicating differentiation rather than pluripotent reprogramming. Scale bars, 50 µm; (**D**) Representative morphology of a chicken iPSC line at passage 40 (P40). Scale bar, 50 µm; (**E**) qRT-PCR analysis of endogenous pluripotency gene expression in P40 chicken iPSCs. Data represent mean ± s.d. of three biological replicates; (**F**) qRT-PCR analysis of in vitro differentiation of chicken iPSCs into the three germ layers. Data represent mean ± s.d. of three biological replicates; (**G**) Immunostaining of chicken iPSCs differentiated cells showing expression of lineage-specific markers: ectoderm (Tuj-1), mesoderm (MHC), and endoderm (Gata4). Scale bar, 50 µm; (**H**) Karyotype analysis of a chicken iPSC line at passage 30 (P30).

Based on our RNA-seq analysis, we selected Oct4, Sox3, Klf4, Myc, Nanog, and Lin28B as core reprogramming factors. Although Sox2 was not enriched in cESCs, we included it in the cocktail given its established role in mammalian reprogramming and its use in previous ciPSC studies [2,3,6,11,12,13,14,25,26]. These seven chicken transcription factors (T7: Oct4, Sox2, Sox3, Klf4, Myc, Nanog, and Lin28B) were delivered to CEFs via seven individual retroviral vectors. Morphological changes were first observed at day 3 after transduction, and iPSC-like colonies emerged by day 5. Large colonies were picked and reseeded onto feeder layers after day 7 (Figure 2A). To assess the contribution of each factor, we systematically removed individual components from the T7 cocktail. While iPSC-like colonies emerged under all single-factor dropout conditions, each omission reduced colony number relative to the complete T7 cocktail (Figure 2B). Moreover, only colonies lacking either Sox2 or Lin28B could be expanded after the first passage, while colonies generated without Oct4, Klf4, c-Myc, or Sox3 could not be sustained beyond several passages. Notably, omission of Sox3 caused colonies to differentiate into neuron-like cells (Figure 2C), further supporting its role as the primary SoxB1 factor in chicken pluripotency. The complete T7 cocktail produced the highest reprogramming efficiency and the most robust expandable colonies and was therefore used for all subsequent experiments.

We next characterized the T7-derived ciPSCs to assess their pluripotency. ciPSCs generated with the full T7 cocktail could be stably maintained for over 40 passages while retaining typical ESC-like morphology and expressing core pluripotency genes (Figure 2D,E). To assess their differentiation capacity, we induced embryoid body (EB) formation and detected lineage-specific markers for the three germ layers: ectoderm (Tuj-1), mesoderm (MHC), and endoderm (Gata4) by immunostaining and qRT-PCR (Figure 2F,G). Karyotyping analysis of one ciPSC line revealed that most cells were diploid with a normal ZZ karyotype, while a small proportion displayed Z chromosome trisomy (Figure 2H). To further assess the molecular identity of the reprogrammed cells, we performed RNA-seq on chicken iPSCs generated using the T7 transcription factor cocktail. Principal component analysis (PCA) of bulk RNA-seq data from ciPSCs, cESCs, and CEFs revealed a close transcriptional relationship between ciPSCs and cESCs (Figure S2A). Heatmap analysis of the top 1000 interquartile range (IQR) genes further confirmed this finding: ciPSCs clustered tightly with cESCs and closely resembled stage X embryos, the developmental stage from which cESCs are typically derived (Figure S2B). Together, these results demonstrate that T7-derived ciPSCs are stably self-renewing, karyotypically normal, and transcriptionally similar to chicken ESCs, indicating successful reprogramming to a pluripotent state.

### 3.4. ciPSCs Contribute to Somatic Lineages in Chimeric Embryos

**Figure 3:**
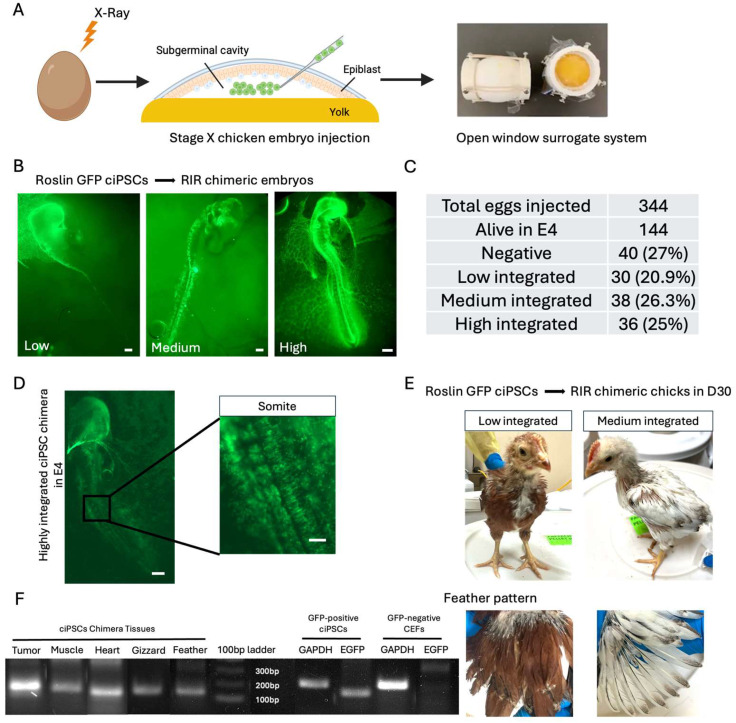
Chimera formation of chicken iPSCs. (**A**) Schematic illustration of the EGK.X blastodermal injection procedure. Recipient embryos were exposed to X-ray irradiation, and GFP-positive ciPSCs were injected into the subgerminal cavity beneath the epiblast at Stage X embryo. The manipulated embryos were then cultured with an open window surrogate system for further development; (**B**) Representative images showing low, medium, and high levels of ciPSC integration in E4 chimeric embryos. Scale bars, 500 µm; (**C**) Quantification of survival rate and integration ratio of chicken iPSC-derived chimeras at E4; (**D**) Highly integrated chimeric embryos generated from ciPSCs. Scale bars, 500 µm (embryo) and 200 µm (somite); (**E**) Photographs of chimeric chicks at ~30 days post-hatch, displaying stable, region-specific patterns of white and colored feathers; (**F**) **Right**: PCR analysis of genomic DNA from GFP-positive ciPSCs and GFP-negative CEFs, showing EGFP amplification alongside GAPDH controls. **Left**: PCR analysis of genomic DNA from various tissues of the hatched ciPSCs chimera chicken.

We next evaluated the developmental potential of ciPSCs by generating chimeras both ex ovo and in ovo. GFP-labeled ciPSCs implanted onto ex ovo EGK.X chicken embryos showed robust integration after 3 days of incubation (Figure S3A). Chimeric embryos were also generated in ovo by injecting GFP-positive ciPSCs into the blastodermal cavity of irradiated EGK.X embryos using a surrogate open-shell system, as previously described [20,31] (Figure 3A). Injected ciPSCs integrated broadly into both somatic and extra-embryonic lineages of the recipient embryos. Integration efficiency was examined at E4 using fluorescence microscopy (Figure 3B), and donor cell contributions were also detected at later developmental stages in the eyes, heart, and gut (Figure S3B). A summary of survival and integration rates at E4 is shown in Figure 3C. GFP signals were detected in every part of the embryo in a highly integrated chimera (Figure 3D). Although many embryos died at E4 due to irradiation, approximately 25% of surviving embryos exhibited high levels of donor cell integration, indicating that ciPSCs possess robust engraftment capacity and developmental potential in vivo.

The overall hatching rate of ciPSC-derived chimeras was low, primarily due to the instability of the open-window incubation system and the pre-irradiation treatment of recipient embryos. Tumor formation was frequently observed in highly integrated chimeras, typically developing in the cranial region and causing hatching failure around embryonic day 19 (Figure S3C). The tumors were likely attributable to residual expression of transgenes, including Sox3 and Nanog, which remained detectable in P40 ciPSCs (Figure S3D). Nevertheless, one low-integration and one medium-integration chimera successfully hatched following the injection of Roslin Green ciPSCs into Rhode Island Red embryos (Figure 3E). The medium-integration chimera died at day 60, and necropsy revealed a stomach tumor. Tissues were collected for PCR analysis, and GFP was detected in multiple tissues, including muscle, heart, gizzard, and feather follicles (Figure 3F). This high level of somatic and extra-embryonic chimerism provides functional evidence that T7-derived ciPSCs possess genuine pluripotency in vivo.

### 3.5. ciPSC-Derived PGC-like Cells Exhibit Limited Germline Competence

**Figure 4:**
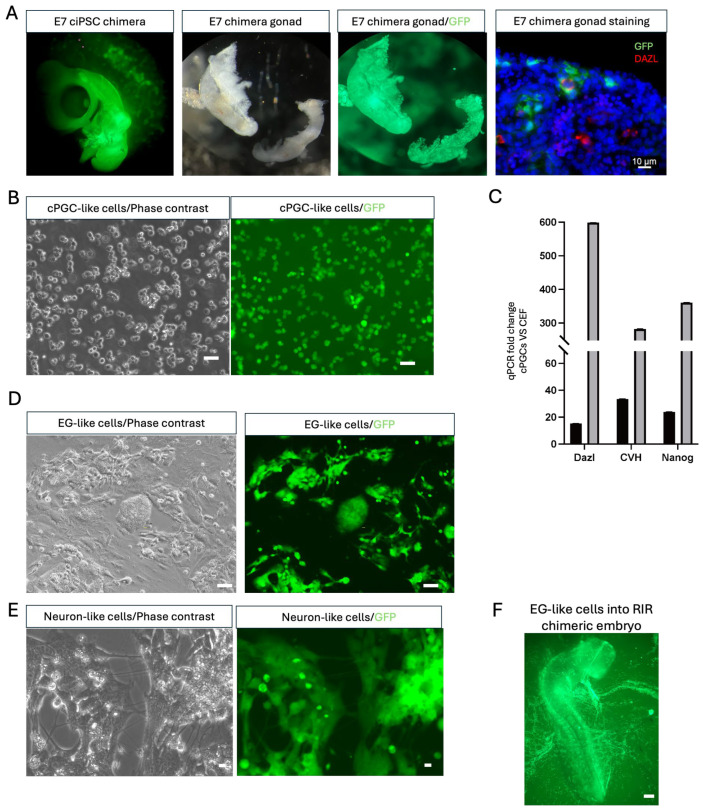
Germline transmission of chicken iPSCs. (**A**) Gonads were isolated from E7 ciPSC-derived chimeric embryos. GFP fluorescence was detected in both left and right gonads under a fluorescence microscope. Immunostaining of gonadal cryosections revealed DAZL- and GFP-double-positive cells. Scale bar, 10 µm; (**B**) GFP-positive PGC-like cells were expanded in FAOT chicken PGC culture medium following fluorescence-activated cell sorting. Scale bar, 50 µm; (**C**) qRT-PCR analysis of germ cell–specific marker expression in GFP-positive PGC-like cells and wild-type (WT) PGCs. Data represent mean ± s.d. of three biological replicates; (**D**) EG-like cells derived from GFP-positive PGC-like cells cultured under OT/3i/chLIF chicken ESC conditions. Scale bar, 50 µm; (**E**) GFP-positive PGC-like cells became neuron-like cells after plating to feeder layers with a change to OT/3i/chLIF chicken ESC culture condition. Scale bar, 25 µm; (**F**) Representative image of a chicken chimeric embryo generated by injection of EG-like cells converted from GFP-positive PGC-like cells. Scale bars, 500 µm.

Having established that ciPSCs can contribute to somatic lineages in chimeric embryos, we next assessed their germline potential. We examined the gonads of E7 ciPSC chimeras and confirmed the presence of DAZL and GFP double-positive cells by immunostaining, indicating donor-derived contribution to the germline lineage (Figure 4A). Using established chicken primordial germ cell (PGC) culture conditions [24,32,33], we isolated and expanded GFP-positive PGC-like cells from the gonads of E6–7 chimeric embryos (Figure 4B). These cells expressed PGC-specific markers, though at lower levels than wild-type PGCs (Figure 4C). Only early-passage PGC-like cells could be converted into embryonic germ (EG) cells using the OT/3i/chLIF culture system, albeit with low efficiency (Figure 4D), as the majority of GFP-positive PGC-like cells differentiated into neuron-like cells when plated on feeder layers (Figure 4E). The successfully converted EG cells retained pluripotency, as demonstrated by their ability to generate highly integrated chimeras (Figure 4F).

Although GFP-positive PGC-like cells were detected in the gonadal region of ciPSC-derived chimeric embryos, their functional identity as bona fide primordial germ cells (PGCs) could not be confirmed. To assess their germline potential, GFP-positive PGC-like cells isolated from the gonads of chimeric embryos were injected into the circulation of E3 recipient chicken embryos. However, no GFP-positive cells were subsequently detected in the recipient gonads. Because authentic PGCs are capable of migrating through the circulation and efficiently colonizing the developing gonads, these results suggest that the ciPSC-derived PGC-like cells did not acquire the full functional properties of germline-competent PGCs. One possible explanation is that the current ciPSC state, or cellular changes acquired during reprogramming and prolonged culture, may restrict complete germline specification and maturation. Collectively, these findings indicate that T7-derived ciPSCs can generate cells expressing germ cell-associated markers and localizing to the gonadal region, but additional optimization will be required to produce fully functional, germline-competent PGCs.

### 3.6. Extension of the T7 Reprogramming System to Other Avian Species

**Figure 5:**
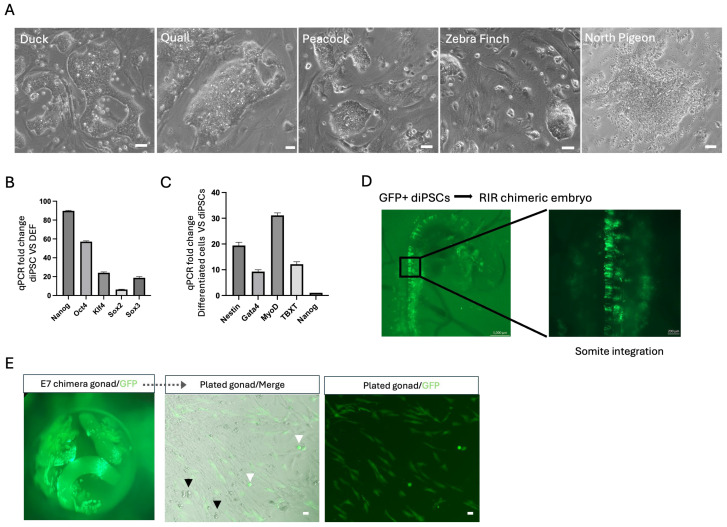
Application of the T7 reprogramming system to other avian species and interspecies chimera formation. (**A**) Representative images of iPSCs generated using the T7 transcription factor cocktail from multiple avian species. Scale bar, 50 µm; (**B**) qRT-PCR analysis of pluripotency gene expression in P30 duck iPSCs. Data represent mean ± s.d. of three biological replicates; (**C**) qRT-PCR analysis of in vitro differentiation of duck iPSCs into the three germ layers. Data represent mean ± s.d. of three biological replicates; (**D**) Images of duck–chicken interspecies chimeras generated by injecting GFP-labeled duck iPSCs into EGK.X stage chicken embryos. GFP-positive duck iPSCs integrated into various regions of the recipient embryos, including the head; (**E**) Images showing the integration of GFP-positive duck iPSCs into the recipient chicken embryo’s gonads. And the isolation and plating of gonads from duck–chicken interspecies chimeras. White arrows indicate GFP-positive PGC-like cells; black arrows indicate GFP-negative PGC-like cells. Scale bar, 50 µm.

To determine whether the T7 reprogramming strategy is broadly applicable beyond chickens, we applied the same system to fibroblasts from a range of avian species. iPSCs were successfully generated from both flightless or ground-dwelling birds (quail, duck, and peacock) and flying species (zebra finch and pigeon) (Figure 5A). Long-term cultured duck iPSCs expressed core pluripotency markers and retained the capacity to differentiate into derivatives of all three germ layers (Figure 5B,C), confirming that stable reprogramming was achieved.

We next tested whether avian iPSCs could contribute to interspecies chimeras. GFP-labeled duck iPSCs injected into chicken EGK.X-stage embryos integrated into the developing embryos, forming interspecies chimeras (Figure 5D). Donor-derived cells were detected in the recipient chicken gonads at E7, and GFP-positive PGC-like cells were identified upon isolation and plating of the gonadal tissue (Figure 5E). However, these PGC-like cells could not be stably expanded, likely due to the lack of a defined culture system for maintaining duck germline cells. Together, these results demonstrate that the T7 reprogramming system generates bona fide iPSCs in chicken and duck and supports iPSC-like colony formation across diverse avian species, suggesting a broadly adaptable strategy for avian iPSC research and future germline preservation.

## 4. Discussion

Previous attempts to generate ciPSCs produced colonies that could not be stably expanded or form highly integrated chimeras [10,11,12,13,14,15]. These failures likely reflect a combination of suboptimal transcription factor cocktails and the absence of culture conditions capable of supporting avian pluripotent self-renewal. By combining the T7 cocktail with OT/3i/chLIF and an avian-adapted E4 basal medium, we overcame both barriers simultaneously. While it is difficult to fully disentangle the contributions of factors and culture, the cultural system was likely a critical and underappreciated variable. In our earlier work deriving cESCs, we found that N2B27, which was originally formulated for neuronal culture, contains additives that can promote neural differentiation when self-renewal signals are insufficient [22]. The E4 medium retains only the four components essential for pluripotent cell proliferation and eliminates these pro-neural cues [21]. Some of the reprogramming failures in earlier ciPSC studies may therefore have been driven by culture conditions promoting differentiation of partially reprogrammed cells, not solely by the choice of transcription factors.

A central finding of this study is the predominant role of Sox3 in avian reprogramming. In mammals, Sox2 is the canonical SoxB1 factor within the pluripotency network [34]. In chickens, however, Sox3 is highly expressed in both ESCs and EGK.X embryos, whereas Sox2 is barely detectable [27]. Removal of Sox3 from the T7 cocktail caused reprogrammed colonies to differentiate into neuron-like cells, whereas removal of Sox2 reduced colony formation efficiency but did not prevent subsequent expansion. Notably, this apparent neural default state was consistently observed across multiple experimental contexts, including OSKM-only reprogramming, Sox3 omission, and the culture of PGC-like cells on feeder layers. These observations suggest that Sox3 plays a critical role in establishing and maintaining pluripotency during avian reprogramming, whereas, in its absence, Sox2 may promote neural lineage commitment, consistent with its well-established function in neural progenitor specification across vertebrates [34]. Nevertheless, whether Sox2 and/or Sox3 are required for the long-term maintenance of established avian pluripotent stem cells remains unclear and will require future loss-of-function studies. The broader requirement for seven factors, compared to four in mammals, suggests a more distributed pluripotency network in birds. No single factor was entirely dispensable in our dropout experiments, yet the contributions were graded: Nanog and Lin28B, which enhance but are not required for mammalian OSKM reprogramming [29], appear to play a more integral role in the avian context, affecting both the timing and robustness of colony formation.

The chimera and germline experiments illustrate both the potential and the current limitations of this system. ciPSCs contributed broadly to somatic and extra-embryonic lineages, with approximately 25% of surviving embryos showing high-level integration at E4, and GFP detected in multiple tissues of hatched chimeras. Donor-derived cells also reached the gonads, where we detected DAZL/GFP double-positive cells and isolated PGC-like cells. However, we did not achieve germline transmission to offspring. The PGC-like cells expressed germline markers at lower levels than wild-type PGCs, were not detected migrating to the gonads via the circulatory system and predominantly adopted neuronal fates when cultured on feeders. Low hatching rates and tumor formation in highly integrated chimeras (typically cranial, around E19) compounded these challenges. Several experimental factors contributed to embryo loss, including pre-irradiation of recipients, the open-shell incubation system, and the use of a donor–recipient strain combination previously associated with reduced chimerism [20]. But the thread connecting the germline deficiency, the neuronal default of PGC-like cells, and the tumor formation most likely traces back to residual retroviral transgene expression. Persistent overexpression of Sox family factors could directly interfere with PGC specification and migration while also predisposing highly integrated chimeras to transformation. Transitioning to non-integrating delivery systems, whether Sendai virus, episomal vectors, or mRNA-based reprogramming [35], is therefore the single most important next step for this platform.

The successful generation of iPSCs from five additional avian species (quail, duck, peacock, zebra finch, and pigeon) using the same T7 cocktail demonstrates that the core transcriptional logic of avian pluripotency is broadly conserved. Duck iPSCs were validated with pluripotency markers, three-germ-layer differentiation, and interspecies chimera formation, while the remaining species are currently supported by colony morphology and expansion data. The duck-to-chicken interspecies chimeras are the most direct proof-of-concept for conservation applications, as donor-derived cells contributed to the host gonads at E7. However, the inability to expand duck PGC-like cells in culture exposes a second major bottleneck beyond transgene removal. Many groups have attempted to develop culture conditions for PGCs from other avian species, but none have achieved cultures as robust and mature as those established for chicken PGCs [36,37,38,39]. Applying this platform to endangered species will therefore require both transgene-free iPSCs and species-specific PGC culture conditions. Realizing this potential will also depend on a deeper understanding of donor–host compatibility within the gonadal niche, particularly whether host-derived signaling cues can support donor PGC maturation and gametogenesis across species boundaries. Together with the cross-species developmental compatibility observed among avian species, this approach could provide a powerful platform for the conservation of endangered birds [20,36,38,40,41,42].

## 5. Patents

One provisional patent related to this study has been filed.

## Data Availability

Chicken embryonic fibroblast. GSM2735363. chicken embryonic stem cells. GSE254284. chicken embryos from EGK.I to EGK.VIII. GSE86592; chicken embryos at HH4. SRX540095 (Weizmann Institute, Israel). The raw RNA-seq data generated for the gene expression profiling analysis of chicken iPSCs, established chicken ES cell lines, chicken embryonic fibroblasts, and primary EGK.X chicken embryos will be deposited in a public repository. Prior to public release, the data are available from the corresponding author upon request.

## Supplementary Materials

The following supporting information can be downloaded at: https://www.mdpi.com/article/10.3390/cells15121092/s1, Figure S1: (A–D) Optimization the medium for chicken iPSCs reprogramming. Summary of culture conditions used during optimization of chicken pluripotent stem cell culture and reprogramming. And representative phase-contrast images of colonies under different culture conditions. Scale bars, 50 µm. Figure S2: Bulk RNA-seq analysis of T7 ciPSCs. (A) Principal component analysis (PCA) of RNA-seq data from CEFs, chicken ESCs, and chicken iPSCs; (B) Heatmap of the top 1000 interquartile range (IQR) genes comparing EGK.X embryos, CEFs, chicken ESCs, and chicken iPSCs. Figure S3: T7 ciPSCs chimera contribution under ex ovo and in ovo. (A) Representative images of implantation of GFP-positive iPSCs onto ex ovo EGK.X chicken embryo; (B) Images showing isolation and plating of gonads from duck–chicken interspecies chimeras. White arrows indicate GFP-positive PGC-like cells; black arrows indicate GFP-negative PGC-like cells. Scale bar, 50 µm; (C) Representative images of E19 chick dead with tumor on the head (left) and E20 chick dead with tumors on the leg and mouth; (D) qRT-PCR analysis of retroviral transgene expression in P40 chicken iPSCs. Data represent mean ± s.d. of three biological replicates. Table S1. Primer sequences in qRT-PCR.
