# Supplementary material for: Generation of Chimera-Competent Avian iPSCs Using Defined Transcription Factors
Source: Cells. 2026 Jun 16;15(12):1092. doi: 10.3390/cells15121092 (PMC13296642; doi:10.3390/cells15121092)
Supplement: Supplementary file 1 [file cells-15-01092-s001.zip › cells-4318118-supplementary.pdf]

Condition	Condition A	Condition B	Condition C	Condition D (AC medium)
Basal Medium	N2B27	N2B27	E4	E4
Go6983	✓	✓	✓	✓
IWR-1	✓	✓	✓	✓
SB431542	✓	✓	✓	
cLIF	✓	✓	✓	✓
CP-673451		✓	✓	✓
Morphology	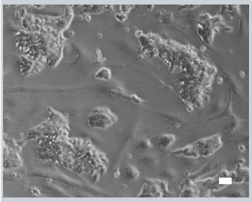	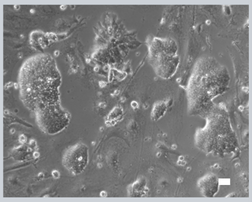	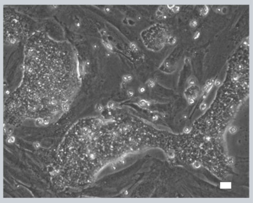	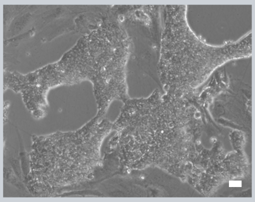

Supplementary Figure S1. (A–D) Optimization the medium for chicken iPSCs reprogramming**.** Summary of culture conditions used during optimization of chicken pluripotent stem cell culture and reprogramming. And representative phase-contrast images of colonies under different culture conditions. Scale bars, 50 μm.

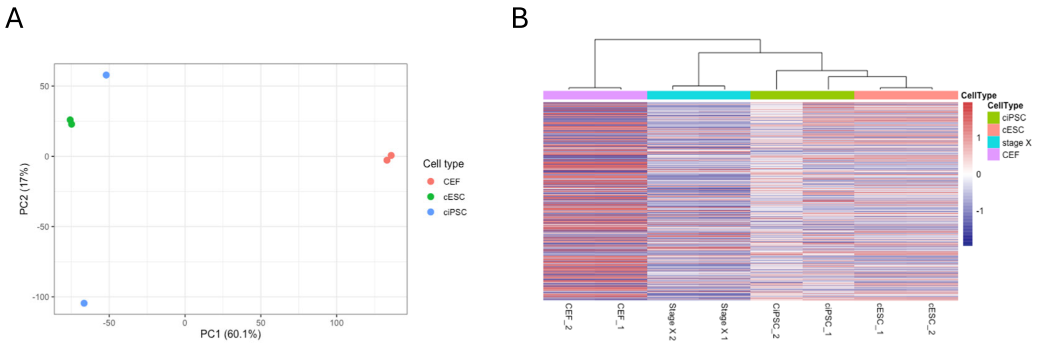

Supplementary Figure S2. Bulk RNA-seq analysis of T7 ciPSCs. (A) Principal component analysis (PCA) of RNA-seq data from CEFs, chicken ESCs, and chicken iPSCs; (B) Heatmap of the top 1,000 interquartile range (IQR) genes comparing EGK.X embryos, CEFs, chicken ESCs, and chicken iPSCs.

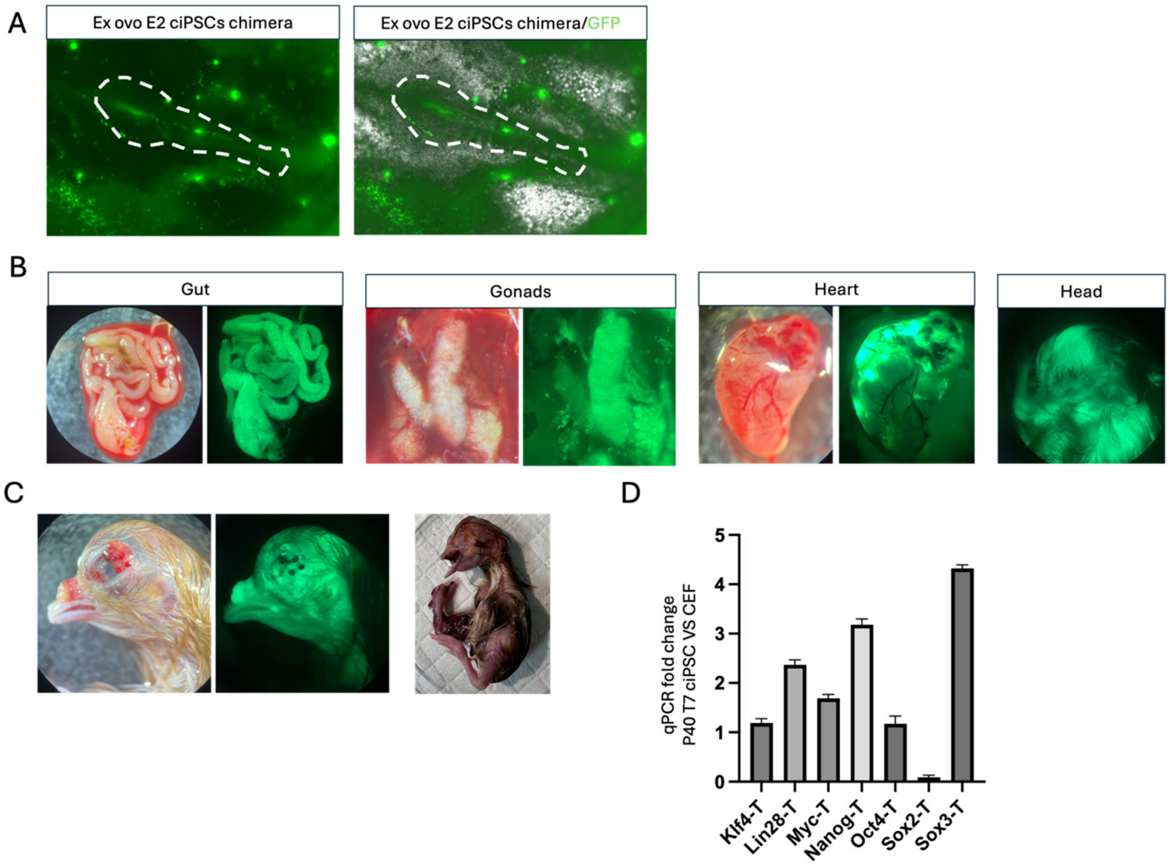

Supplementary Figure S3. T7 ciPSCs chimera contribution under ex ovo and in ovo**.** (A) Representative images of implantation of GFP-positive iPSCs onto ex ovo EGK.X chicken embryo; (B) Images showing isolation and plating of gonads from duck–chicken interspecies chimeras. White arrows indicate GFP-positive PGC-like cells; black arrows indicate GFP-negative PGC-like cells. Scale bar, 50 µm; (C) Representative images of E19 chick dead with tumor on the head (left) and E20 chick dead with tumors on the leg and mouth;(D) qRT-PCR analysis of retroviral transgene expression in P40 chicken iPSCs. Data represent mean ± s.d. of three biological replicates.

	Forward	Reverse
Chicken Primers		
RS17(House keep)	ACACCCGTCTGGGCAACGAC	CCCGCTGGATGCGCTTCATC
Oct4	AGCACAGGAGAGGGGTTG	CAACTACAGCAGGCTCAAAGG
Nanog	CTCCGCTGCCTCTTTTGC	CTGGGCTACAAATAGGGTATTTCTTG
SOX3	ACTGTGAACGATGTTTTGACATATCAG	GGAAGAAATCCGGTAAACAAAACAAAA
Sox2	CCCCTGTGGTTACCTCTTCCTCC	TGCCGTTAATGGCCGTGCC
KLF4	ACTCTGGGTTCCGCTCTTC	CCCACTCTTACCCCGTACTC
CMYC	GATACTTTGGGCATAAGGGATGATG	CTGGGGACAACTCTATTTGGAATTC
Lin28B	GACCATGCGAGCGAAATTGG	CCTTTACTCGCCCCTGCTTC
Nestin	GCAGAGCCAGAGCGCACCAA	CGTGTCACCCCGACTCGGAC
Pax6	AACTCCATCAGCTCCAATGG	AGGGCTTCGATTTGCTCTTG
BMP4	TCCACCATGAAGAGCACCTG	TGAGGTTGAAGACGAAGCGG
TBXT	AGGTCAAGCTCACCAACAAG	AGGAATGGCTGGTGATCATC
MyoD	TCTCGCTCCCTCGAGAAAAA	AGTGCGATTTCCCCAGACAG
Gata4	TTCGACAGCCCCATGCTGCAC	AAATTCGATGTTGGCATGCCGGG
Oct4-T	GACGTGGTGAGAGTGTGGTT	CGTACATCACTCCCTCGCTC
Sox3-T	GGGCCAGAGGATTGACACTT	ATGTCGTAGCGGTGCATCTG
Klf4-T	CACACATGCGATTACGCTGG	GCGAATTTCCATCCGCATCC
cMyc-T	GCGCTGGACTCTATCTGCAT	TGGGTATGGAAACACCACGG
Nanog-T	CACCCATCTCACCCCACTTTT	ACTTCTGGCTCTGAAACCGC
Sox2-T	TGGTCAAGACGGAATCCAGC	GATCATGTCCCGAAGGTCCC
Lin28-T	CTCAGGGAAAGCCTGCCTAC	GGCGGCCGCTCATTCC
Duck Primers		
GAPDH	GAGGGTAGTGAAGGCTGCTG	CATCAAAGGTGGAGGAATGG
Nanog	GGTTTCAGAACCAACGGATG	GTGGGGGTCATATCCAGGTA
Oct4	TGGGCACTCTCTATGGGAAGA	ATTGAGCCAACGCTGTAGCA
Klf4	GAGAACCCACACAGGTGAGAAA	GGATCGGGCAAACTTCCATC
Sox2	CACAACTCCGAGATCAGCAA	TATAATCCGGGTGCTCCTTC
Nestin	GGCGACCTACAGACCCCAC	ACAGCCCAAAAATCCGGGGT
Gata4	GCACGCCAACATCGAATTTTT	CATCTTGTGGTAGAGCCCGC
MyoD	AAG GCG TGC AAG AGG AAG AC	TGG TTG GGG TTG GTG GA
TBXT	ACAACTCGCCAACAGCCTAT	GACTGGAGCCAGTAGCTGTG

Supplementary Table S1. Primer sequences in qRT-PCR.
